# Gene-activation of surface-modified 3D printed calcium phosphate scaffolds

**DOI:** 10.1186/s13065-025-01390-9

**Published:** 2025-02-21

**Authors:** Noah Z. Laird, Pornpoj Phruttiwanichakun, Esraa Mohamed, Timothy M. Acri, Leela R. Jaidev, Aliasger K. Salem

**Affiliations:** 1 Department of Pharmaceutical Sciences and Experimental Therapeutics, College of Pharmacy, University of Iowa, 180 S Grand Ave, Iowa City, IA 52242 USA; 2 Pharmaceutics Department, Faculty of Pharmacy, Assiut University, Assiut, Egypt; 3 Department of Periodontics, College of Dentistry and Dental Clinics, University of Iowa, Iowa City, IA USA

**Keywords:** Gene delivery, Biomaterials, Bone, Tissue engineering, Regenerative medicine, 3D printing

## Abstract

Large volume bone defects that do not spontaneously heal despite surgical stabilization (“critical-sized” defects) remain a challenge to treat clinically. Recent research investigating bone regenerative implants made from 3D printed materials have shown promise as a potential alternative to current treatment methods, such as autografting, allografting, and multi-step surgical interventions. Recent work has shown that implanting 3D printed calcium phosphate cement (CPC) scaffolds loaded with bone morphogenetic protein-2 (BMP-2) can provide a one-step surgical intervention that has similar bone healing outcomes to a popular two-step intervention: the Masquelet technique. The aim of this study was to investigate whether a 3D printed CPC scaffold loaded with a lyophilized polyplex gene-delivery formulation could serve as an alternative to loading BMP-2 protein onto such scaffolds. We 3D printed CPC scaffolds, hardened them with multiple methods, and explored the impact of these hardening methods on surface texture, mechanical strength, osteogenic differentiation, and ion flux. We then gene-activated these materials with cationic polyplexes containing plasmid DNA encoding reporter genes to investigate transfection from the gene-activated scaffolds. We found that incubating CPC scaffolds in aqueous solutions after initial hardening in a humid environment could enhance scaffold mechanical strength (compressive strength of 21.28 MPa vs. 6.54 MPa) and osteogenic differentiation. We also found that when we increased the total surface area of the CPC material exposed to polyplex solutions, there was a reduction in transfection via adsorption of polyplexes to the CPC surface. This study shows that 3D printed, gene-activated CPC scaffolds are a promising avenue for future exploration in the field of bone regeneration, though the level of gene expression induced by the scaffolds must be improved.

## Introduction

Bone tissue has a limited ability to heal itself naturally after injury, though this healing ability is insufficient if a bone defect is large enough. Bone defects that will not spontaneously heal despite surgical stabilization are termed “critical-sized defects”, and remain a challenge to treat clinically [1–3]. These critical defects can be caused by clinical situations including infection requiring bone debridement, resection of bone tumors, congenital deformities, high energy traumas, and blast injuries [2–4]. These types of injuries are typically treated with autografts, allografts, or implanted materials intended to replace or regenerate missing bone [3]. These approaches can be successful in healing some critical sized defects, but challenges remain because autograft volume is limited, non-regenerative implants tend to fail over time, and allografts carry a risk of disease transmission [3, 5–7]. Large segmental defects have historically had poor outcomes that often required amputation, though more recent techniques (such as the induced membrane, or “Masquelet”, technique) have had more success [3, 8]. Recent research in the field of tissue engineering has investigated new implant materials that aim to match the bone healing of autografts while avoiding the issues of graft rejection, disease transmission, and limited graft availability [9].

Researchers in the field of bone tissue engineering often use a biomaterial scaffold to induce and/or guide bone regeneration. Through their work they found that endowing a scaffold with a rough exterior surface [10–13], an interconnected pore network [14–16], and pore sizes of 200-400 µm [17–20] throughout the volume of the scaffold can improve bone regeneration in scaffolds regardless of the material used. 3D printing has become popular within tissue engineering because the technique allows precise control over material composition and morphology [21]. In fact, 3D printing can enable researchers to dictate the structure of the internal pore network within their scaffolds by adjusting the pore diameter, pore density, and pore shape in the software used to design their scaffolds. An additional benefit to 3D printing is that a scaffold can be designed to perfectly replicate most 3D shapes, and 3D medical imaging techniques can be combined with 3D printing to create scaffolds that perfectly match a patient’s own morphology [22]. This means that a 3D printed bone regenerative therapeutic might be used in craniofacial reconstruction and plastic surgery to regenerate bone that exactly replicates the patient’s original bone structure or even amends a pre-existing congenital defect.

One popular technique for the treatment of large diaphyseal bone defects is the Masquelet technique. In this technique the defect is first filled with a spacer for 6–8 weeks to induce a membrane to form around the defect, after which the spacer is replaced with bone graft materials to regenerate the lost bone [8]. While this technique has had success [23], it requires patients to undergo two distinct surgical procedures, which can cause them additional pain and increase the total cost of healing. Recent work has suggested that a 3D printed calcium phosphate implant loaded with a bone regenerative protein, bone morphogenetic protein 2 (BMP-2), might be a one-step alternative to the two-step Masquelet technique [24]. In their study, Fenelon et al. found that their BMP-2-loaded calcium phosphate scaffolds implanted in a single procedure provided a comparable degree of bone defect healing to that achieved by the two-step Masquelet technique [24]. BMP-2 protein is already used in the clinic to enhance bone regeneration [25, 26]. BMP-2 protein itself is very potent and does indeed enhance bone healing, but the local administration of the protein by loading it onto scaffolds can be problematic [27–30]. In order to maintain a therapeutic concentration of BMP-2 protein at the injury site, large amounts of the protein must be loaded onto the scaffold to combat the dilution, degradation, and aggregation that all proteins experience in vivo [31]. The large doses required mean that therapeutics containing BMP-2 protein are very expensive and they can lead to off-target effects, including ectopic bone formation in local non-bone tissue [28–30, 32–34]. Some research has shown that loading scaffolds with BMP-2 protein contained within sustained release systems can improve bone healing, however, this approach still relies upon production of BMP-2 using expensive biological manufacturing processes [35–37]. One alternative to loading proteins onto implant materials to enhance tissue regeneration is to “gene-activate” the materials by loading them with nucleic acids to induce expression of therapeutic proteins in cells local to the implanted material [38, 39]. Researchers have shown that incorporating a gene delivery system into scaffolds can increase their functionality by inducing cells local to the scaffold and/or cells that invade the scaffold to produce therapeutic proteins that enhance healing outcomes [40–42]. Prior work has demonstrated the effectiveness of including a gene delivery system that induces expression of bone morphogenetic protein 2 (BMP-2), among other osteogenic proteins [43–52].

The aim of this study was to investigate the loading of a gene-delivery system as a lyophilized formulation into surface modified 3D printed calcium phosphate materials to begin exploring their potential as gene-activated scaffolds for bone regeneration. To the authors’ knowledge, this work is the first attempt to use a lyophilized gene-delivery system loaded onto a 3D printed scaffolds for bone regeneration. We began by 3D printing a well-characterized commercial calcium phosphate cement (CPC) and modifying the hardening methods to induce a textured surface, which is known to improve bone healing outcomes. We then explored gene-activating the prints, lyophilizing them, and finally assessing their transfection and gene expression profiles using a gold standard non-viral gene delivery system; polyethylenimine (PEI) complexed with plasmid DNA (pDNA) to create PEI-pDNA polyplexes.

## Materials and methods

### Printing of CPC scaffolds

Non-aqueous CPC was purchased from Innotere GmbH (Radebeul, Germany) and loaded into 3 mL printing cartridges (Nordson EFD, Westlake, OH, USA) affixed with a conical needle tip (0.2 mm internal diameter or 0.437 mm internal diameter) (Fisnar, Germantown, WI, USA). The cartridges were loaded into a BioX 3D printer (Cellink, Gothenburg, Sweden), and printed using a 3 mL pneumatic printhead (Cellink). Printing was performed at room temperature with printing pressures of 100–700 kPa and printing speeds of 3–10 mm/s. All scaffold designs were created using 3D Builder (Microsoft, Redmond, WA, USA) and sliced using Slic3r (version 1.3.0, open source). CPC discs were printed with a rectilinear infill pattern with 100% infill, CPC 3D lattices were printed with a rectilinear infill pattern with 50% infill. Surface area of CPC discs was determined by calculating the surface area of a cylinder with the same dimensions as the disc (7.5 mm diameter, 1 mm height). Surface area for CPC lattices was determined by calculating the surface area for each layer, then multiplying by the number of layers [7]. The surface area for each layer was calculated by determining the length of rectilinear infill pattern, then calculating the surface area of a cylinder with the same dimensions (23.3 mm length, 0.437 mm diameter).

### Hardening and texturing of CPC scaffolds

After printing, scaffolds were either immersed in ultrapure water at room temperature for 3 days (“water alone method”, or “WA”) or placed in a humidified incubator at 37 °C for 3 days (“vapor alone method”, or “VA”). After the incubation, scaffolds were washed 3 times with acetone (20 min of immersion in acetone per wash), then left to dry at room temperature. Some of the scaffolds hardened with the VA method were immersed in either ultrapure water (“vapor + water”, or “VW) or simulated body fluid (SBF) (“vapor + SBF”, or “VS”) for 3 days at room temperature. SBF was prepared by dissolving salts (all from Sigma Aldrich) in ultrapure water (ThermoFisher Scientific) to produce a solution containing 40.286 mM NaCl, 1.143 mM KCl, 0.143 mM MgSO_4_, 0.286 mM MgCl, 1.2 mM NaHCO_3_, 2.5 mM CaCl_2_, and 1 mM KH_2_PO_4_. CPC scaffolds were incubated in the secondary incubation solutions at a volume/weight ratio of 21.52 mL of solution per 1 g of CPC. After the secondary incubations, the acetone washes were repeated before the scaffolds were left to dry at room temperature.

### Scanning electron microscopy

Cylindrical 3D lattice scaffolds (7.5 mm in diameter and 1 mm in height, 50% rectilinear infill pattern, 0.2 mm strand diameter) were printed and hardened as described above. Samples were sputter coated with a gold–palladium alloy and imaged with field emission scanning electron microscopy (Hitachi S-4800, Hitachi, Japan).

### Compression testing

Cylindrical 3D lattice scaffolds (7.5 mm in diameter and 3 mm in height, 50% rectilinear infill pattern, 0.2 mm strand diameter) were printed and hardened as described above. Scaffolds were compressed with an MTS Insight Material Testing System (MTS Systems, Eden Prairie, MN, USA) loaded with a 1kN load cell at a rate of 1 mm/min until failure. The resulting stress vs. strain plots were used to calculate compressive strength and Young’s modulus.

### Specific surface area analysis

Rods 0.2 mm in diameter and 2 mm long were printed and hardened as described above. Rods were divided into sample groups of equal weight, then analyzed with a Beckman-Coulter surface analyzer (Model SA-3100, Miami, FL). N_2_ adsorption isotherms at 77 K were collected over a relative pressure range of 0.00 to 0.20 in 0.02 increments. The Brunauer, Emmett, and Teller (BET) theory was applied to five points in the relative pressure range of 0.00 to 0.20. All data analyses are built into the SA-3100 analyzer.

### Calcium content and pH after incubation

Discs (7.5 mm in diameter, 1 mm in height, 100% rectilinear infill pattern) were printed and hardened as described above. Each disc was incubated in 0.5 ml of Dulbecco’s Modified Eagle Medium (DMEM) containing 1% sodium pyruvate, 1% HEPES buffer, 1% GlutaMAX^™^ (all from ThermoFisher Scientific, Waltham, MA, USA), 0.05 mg/ml gentamycin sulfate (IBI Scientific, Dubuque, IA, USA), and 10% fetal bovine serum (FBS, Atlanta Biologicals, Flowery Branch, GA, USA) (termed “complete DMEM”) in a humidified incubator for 24 h. Medium was collected and aliquots were removed for pH measurement (S20 SevenEasy, Mettler Toledo, Columbus, OH, USA). Calcium content was assessed with a commercial calcium colorimetric assay kit (MAK022, Millipore Sigma, Burlington, MA, USA) according to the manufacturer’s protocol.

### Alkaline phosphatase activity assay

Discs (7.5 mm in diameter, 1 mm in height, 100% rectilinear infill) were printed and hardened as described above. Each disc was seeded with 12,500 bone marrow-derived mesenchymal stem cells (BMSCs) obtained from the American Type Culture Collection (ATCC). Discs and cells were incubated for 7 days, after which an alkaline phosphatase activity assay (ab83369, Abcam, United Kingdom) was used according to the manufacturer’s protocol to assess the activity in the cell lysate. Total DNA from the cell lysate was measured via a Quant-iT^™^ PicoGreen^™^ assay (ThermoFisher Scientific) according to the manufacturer’s protocol and was used to normalize the enzyme activity data.

### Purification of plasmid DNA

Plasmid DNA was purified from DH5α *Escherichia coli* that had been previously transformed with plasmids encoding either Enhanced Green Fluorescent Protein (EGFP) (Plasmid #13031, AddGene, Watertown, MA, USA) or *Cypridina* luciferase (Cat. #16150, ThermoFisher Scientific). Purification was performed using a GenElute^™^ HP Endotoxin-Free Plasmid Maxiprep Kit (Sigma-Aldrich, St. Louis, MO, USA) according to the manufacturer’s protocol.

### Polyplex solution preparation

Polyplexes were prepared as described previously [53]. Briefly, two 500 μl solutions containing either 130 μg of 25-kDa branched PEI (Sigma-Aldrich) or 100 μg of pDNA were prepared in DNAse/RNAse-free water (ThermoFisher Scientific). The PEI solution was added to the pDNA solution, vortexed for 30 s, and incubated for 30 min to allow for complexation between the pDNA and PEI. The resulting 1 ml polyplex solution had a nitrogen (N) to phosphate (P) ratio (N/P ratio) of 10. To prepare polyplex solutions containing 2% (w/v) sucralose, 111.1 μl of 20% (w/v) sucralose (Sigma-Aldrich) in DNAse/RNAse-free water (ThermoFisher Scientific) were added to the polyplex solution (final pDNA concentration of 90 μg/ml).

### Cell culture

HEK 293 T cells and MC3T3 cells (MC3T3-E1, subclone 4) were obtained from the American Type Culture Collection (ATCC, Manassas, VA, USA). HEK 293 T cells were cultured in DMEM containing 1% sodium pyruvate, 1% HEPES buffer, 1% Glutamax (all from ThermoFisher Scientific), 0.05 mg/ml gentamycin sulfate (IBI Scientific, Dubuque, IA, USA), and 10% fetal bovine serum (FBS, Atlanta Biologicals, Flowery Branch, GA, USA) in a humidified incubator at standard culture conditions (37 °C and 5% CO_2_, Sanyo Scientific, Japan). MC3T3 cells were cultured in complete Minimum Essential Medium Alpha (MEM Alpha) containing 1% penicillin–streptomycin (ThermoFisher Scientific) and 10% FBS (Atlanta Biologicals). Cells were passaged with 0.25% trypsin–EDTA (ThermoFisher Scientific).

### Gene-activation of CPC discs and lattices with lyophilized coating

CPC discs (7.5 mm diameter, 1 mm height, 100% rectilinear infill, calculated surface area = 111.92 mm^2^) and cylindrical CPC 3D lattices (4.8 mm diameter, 2.8 mm height, 50% rectilinear infill, 0.437 mm strand diameter, calculated surface area = 225.97 mm^2^) were printed and hardened with the VS method, as described above. For gene-activation of CPC discs, polyplex solutions containing 2% (w/v) sucralose were prepared as described above, then 13.89 μl of polyplex solution containing 1.25 μg of pDNA were pipetted onto the discs. Polyplex-loaded discs were then frozen at − 80 °C and lyophilized (FreeZone 4.5 − 105, Labconco, Kansas City, MO, USA). For gene-activation of CPC 3D lattices, lattices were placed into 96-well plates containing 100 uLs of 2% (w/v) sucralose in pure water, then exposed to vacuum in a vacuum desiccator to remove air trapped in the nanotexture of the CPC. The lattices were then removed from the wells containing sucralose solution, placed in new 24-well plates, frozen at − 80 °C, then lyophilized overnight (FreeZone 4.5 − 105, Labconco). After the initial lyophilization, 25 µl of polyplex solution (0% sucralose, 100 µg/ml pDNA) were pipetted onto the center of the lattices, after which the lattices were frozen at − 80 °C and lyophilized overnight again (FreeZone 4.5 − 105, Labconco). CPC 3D lattices were printed with such dimensions and infill percentage such that the lattice could be loaded with 25 µl of polyplex solution (containing 2.5 µg of pDNA in polyplexes, prepared as described above).

### Transfection efficiency and mean fluorescence intensity assessment from gene-activated CPC

Cells were seeded onto CPC prints gene-activated with pDNA encoding EGFP. Cells were seeded at a density based on the type of cell (HEK 293 T or MC3T3) and the type of CPC print (disc or lattice). CPC discs were placed in 48-well plates and seeded with 37,500 HEK 293 T cells per well. CPC lattices were placed in 24-well plates and seeded with 75,000 HEK 293 T cells or 20,000 MC3T3 cells per well. Cell seeding was performed in serum-free medium, which was collected and replaced with complete medium after 4 h. Cells were then incubated for 72 h. Transfection efficiency and mean fluorescence intensity was assessed via flow cytometry using a FACScan (Becton Dickinson, Franklin Lakes, NJ, USA) flow cytometer equipped with a 15 mW of 488-nm excitation laser. Forward scatter, side scatter, and EGFP fluorescence (FL1 channel, 560-nm filter) parameters were measured. Cell debris was excluded through analysis with FlowJo^™^ software (version 10, Becton Dickinson). A fluorescence threshold based on the negative control was created, and the percentage of cells fluorescing above the threshold was determined for each sample. To determine mean fluorescence intensity, the intensity in the FL1 channel for all events within a sample was averaged and log transformed (log base 10).

### Cypridina luciferase expression characterization from gene-activated CPC

CPC lattices gene-activated (as described above) with 25 µl of polyplex solution containing 2.5 µg of pDNA encoding secreted *Cypridina* luciferase were seeded with HEK 293 T cells (75,000 cells per well) or MC3T3 cells (20,000 cells per well) in 24-well plates. Cell seeding was performed in serum-free medium, which was collected and replaced with complete medium after 4 h. Conditioned medium was collected and replaced completely every 2 days for 8 days. Luciferase activity in the conditioned media was analyzed with a Pierce^™^ *Cypridina* Luciferase Glow Assay Kit (Cat. # 16170, ThermoFisher Scientific) according to the manufacturer’s instructions using a microplate reader (SpectraMax M5, Molecular Devices, Sunnyvale, CA, USA).

### Adsorption of polyplexes to CPC particulates

Polyplex solutions were prepared as described above and CPC prints were pulverized in a bead mill. The resulting CPC particulates were mixed with 1 ml of polyplex solution for 5 min. The resulting mixture and 1 ml of polyplex solution not containing CPC particulates were then centrifuged for 30 s (10,000 *g*). HEK 293 T cells were seeded onto 24-well plates at a seeding density of 50,000 cells per well 24 h prior to transfection. Cells were treated with non-centrifuged polyplex solution (Polyplexes), the supernatant of centrifuged polyplex solution (Poly. Supernatant), the supernatant of centrifuged CPC/polyplex mixture (CPC/Poly Supernatant), or the resuspended pellet from the centrifuged CPC/polyplex mixture (CPC/Poly Mixture), then incubated for 72 h. Transfection efficiency was assessed with flow cytometry (as described above) and fluorescence microscopy (EVOS FL, ThermoFisher Scientific).

### Statistical analysis

All statistical analysis was performed with Prism (version 9, GraphPad, San Diego, CA, USA). Datasets were assessed with QQ plots, residual normality tests, and homoscedasticity tests to check whether the ANOVA assumptions of equal variance and normal distribution were violated. Figure 3B was analyzed with a Kruskal–Wallis test with Dunn’s multiple comparisons testing due to the dataset violating the assumption of normal distribution. Figures 2B, C and 3A were analyzed with the Brown-Forsythe and Welch test with Dunnett’s T3 multiple comparisons testing due to the datasets violating the assumption of normality. All other statistical analyses used ordinary one-way ANOVA with Tukey’s multiple comparisons tests.

## Results

### Surface analysis of CPC prints

**Fig. 1:**
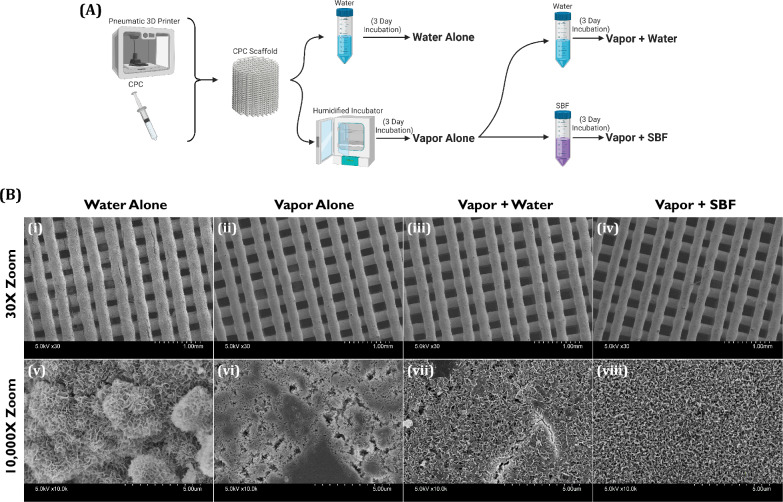
Generation and visualization of differently formulated CPC constructs. **A** Schematic diagram for the preparation of CPC prints hardened with varying methods. CPC prints were generated, then incubated in either water or a humidified incubator to create the “Water Alone” and “Vapor Alone” groups, respectively. Some Vapor Alone prints were then subjected to secondary incubations in either water or simulated body fluid (SBF) to create the “Vapor + Water” and “Vapor + SBF” groups, respectively. **B** SEM images of printed CPC constructs at varying magnifications. Images depict 3D lattices with 0.2 mm strand diameter and 50% infill at 30X magnification (B(i-iv)) and 10,000X magnification (B(v-viii))

**Fig. 2:**
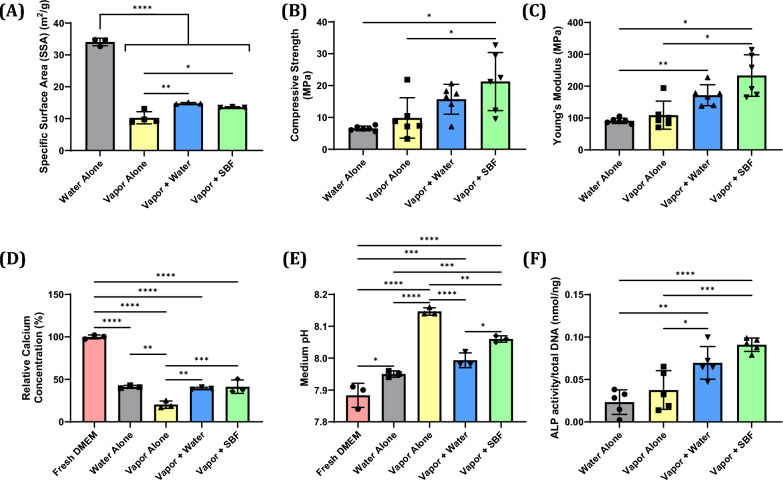
**A** Specific surface area of printed CPC rods (0.2 mm diameter, 4 mm length) as calculated by the Brunauer-Emmet-Teller (BET) method using nitrogen gas as the adsorbate (n = 3–4). **B** Compressive strength and **C** Young’s modulus of CPC 3D lattices printed with 0.2 mm strand diameter, 50% infill, 7.5 mm total diameter, and 3 mm height (n = 6 for all groups). **D** Calcium content and **E** pH of medium incubated with CPC discs for 24 h (n = 3 for all groups). **F** Alkaline phosphatase activity from cell lysate of BMSCs cultured on CPC discs for 7 days normalized to total DNA (n = 5 for all groups). Results are expressed as the mean ± SD where *p < 0.05, **p < 0.01, ***p < 0.001, ****p < 0.0001

CPC prints prepared as shown in Fig. 1 A were imaged via SEM at low magnification (30X zoom) and high magnification (10,000X zoom). Analysis of the low magnification SEM micrographs of CPC prints (Fig. 1B(i-iv)) shows that the WA hardening method induced crack formation in the struts of the CPC lattice while the VA, VW, and VS hardening methods did not. Viewing the CPC prints at high magnification (Fig. 1B(v-viii)) showed clear differences in surface texture, with the VA method showing the least textured surface while the WA, VW, and VS methods showed more textured surfaces than the VA group. These visual observations are supported by the VA method yielding the lowest SSA relative to the other hardening methods (Fig. 2A). The WA group had the highest SSA (34.072 m^2^/g) and the VA group had the lowest SSA (10.259 m^2^/g), while the VW and VS groups were in between (14.8107 m^2^/g and 13.597 m^2^/g, respectively). The VW and VS groups were not significantly different from each other, but they were both significantly different from the WA and VA groups (Fig. 2A).

### Mechanical testing of CPC prints

CPC prints were subjected to a mechanical compression test and the compressive strength and Young’s modulus were calculated. We found that for both compressive strength and Young’s modulus the mean values for each group followed the same pattern: WA < VA < VW < VS (Fig. 2B and C). Only the VS prints were significantly different from both the WA and VA prints in both metrics (with mean compressive strength and Young’s modulus values being 21.28 MPa and 233.3 MPa, respectively). However, the Young’s modulus for the VW prints (mean value of 171.5 MPa) was also significantly different from that of the WA prints.

### Effect of CPC prints on cell culture medium

CPC prints were immersed in DMEM and incubated for 24 h, after which the media’s calcium concentration and pH were assessed. We found that all CPC prints reduced the calcium concentration to less than 50% of that of fresh DMEM and increased medium pH relative to fresh DMEM (Fig. 2D and E). The magnitude of reduction in calcium concentration and increase in pH varied between the CPC hardening methods, with the VA prints having both the greatest decline in calcium concentration (20.25% of fresh DMEM) and the greatest increase in medium pH (pH = 8.147). The WA, VW, and VS methods all had equivalent calcium content (~ 40% of fresh DMEM). The WA and VW groups had elevated pH relative to fresh DMEM (pH = 7.950 and 7.993, respectively), but had a lesser pH than the VS group (pH = 8.060).

### Effect of hardening method on BMSC differentiation

BMSCs were seeded onto CPC prints and incubated for 7 days, after which the cells were lysed and their alkaline phosphatase (ALP) activities were assayed and controlled to total DNA. We found that the ALP activities from CPC prints followed the same pattern as the mechanical testing results: WA < VA < VW < VS (Fig. 2F). Interestingly, while the VW and VS groups were not significantly different from each other and both showed significant differences from the WA and VA groups, the VS group had a greater mean ALP activity than the VW group.

### Characterization of transfection efficiency from CPC discs hardened with different hardening methods

**Fig. 3:**
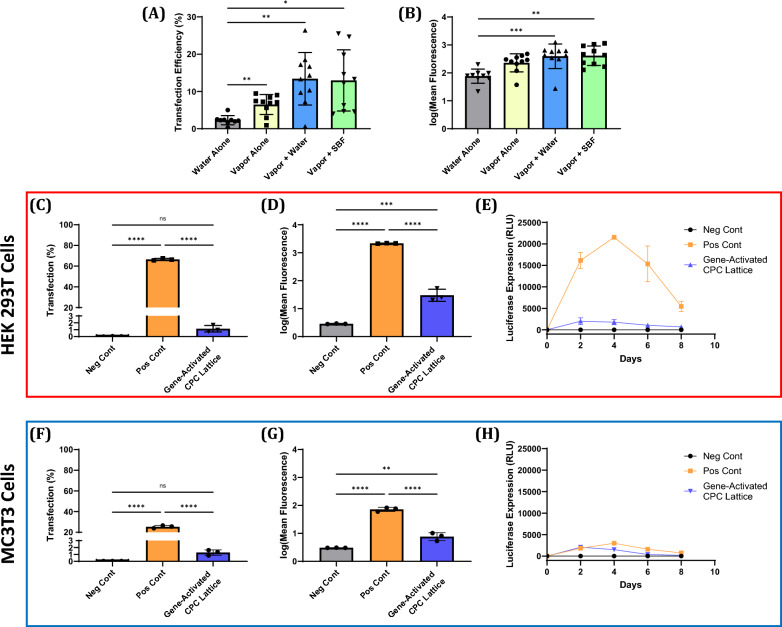
**A** Transfection efficiency and **B** log transformed mean fluorescence intensity of HEK 293 T cells 72 h after being seeded onto gene-activated CPC discs that were hardened via the indicated methods (n = 9–10). **C** Transfection efficiency and **D** log transformed mean fluorescence intensity for HEK 293 T cells 72 h after being seeded onto gene-activated CPC lattices (n = 3). **F** Transfection efficiency and **G** log transformed mean fluorescence intensity for MC3T3 cells 72 h after being seeded onto gene-activated CPC lattices (n = 3). Luciferase gene expression from **E** HEK 293 T cells and **H** MC3T3 cells seeded onto gene-activated CPC lattices and incubated for 8 days (n = 3). Results are expressed as the mean ± SD where ns = no significance, **p < 0.01, ***p < 0.001, ****p < 0.0001

CPC discs hardened via the WA, VA, VW, and VS methods were gene-activated with polyplexes (encoding EGFP) via a lyophilized coating, then seeded with HEK 293 T cells. Transfection efficiency and mean fluorescence were assessed via flow cytometry after 3 days of culture. We found that all gene-activated CPC discs were able to induce expression of the gene of interest, but the VW and VS methods had the highest mean transfection efficiency (13.42% and 12.97%, respectively) and log(mean fluorescence) (2.596 and 2.614, respectively) (Fig. 3A and B). The VW and VS hardening methods also exhibited very high variability in their transfection efficiency, with the standard deviation being greater than half the value of the mean (7.048% and 8.216%, respectively).

### Characterization of gene expression from CPC lattices

CPC lattices hardened with the VS method were gene-activated with polyplexes (encoding either EGFP or luciferase) via a lyophilized coating, then seeded with either HEK293T cells or MC3T3 cells. Lattices gene-activated with pDNA encoding EGFP were seeded and incubated for 3 days, after which the transfection efficiency and log(mean fluorescence) were assessed via flow cytometry. Lattices gene-activated with pDNA encoding luciferase were seeded and incubated for 8 days, during which the luciferase expression was assessed periodically. Both cell types were able to be transfected by gene-activated CPC lattices (Fig. 3D and G), albeit with relatively low transfection efficiencies (1.137% for HEK 293 T cells, 1.273% for MC3T3 cells) (Fig. 3C and F). When the gene expression profile was assessed, both cell types exhibited detectable luciferase expression beginning at day 2 and ending at day 8, roughly matching the total time during which the positive control expressed the gene of interest (Fig. 3E and H). For HEK 293 T cells, the positive control vastly outperformed the gene-activated CPC lattice in terms of luciferase expression, while for MC3T3 cells the positive control displayed only slightly higher gene expression relative to the gene-activated CPC lattice.

### Transfection after adsorption of polyplexes to CPC particulates

**Fig. 4:**
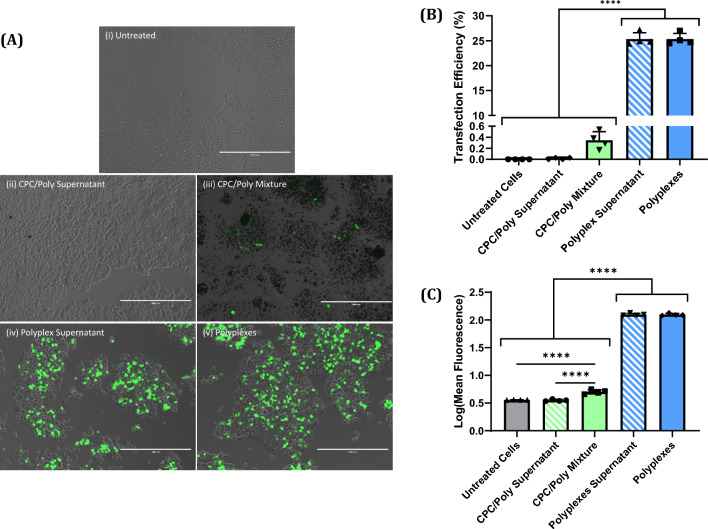
**A** Composite micrographs (fluorescence and brightfield) of HEK 293 T cells treated with either water (Untreated), polyplex solution (Polyplexes), centrifuged polyplex solution (Polyplex Supernatant), CPC powder mixed with polyplex solution (CPC/Poly Mixture), or the supernatant from the centrifugation of the previous mixture (CPC/Poly Supernatant). Green coloration indicates fluorescence from EGFP. All scalebars represent 400 μm in length. **B** Transfection efficiency and **C** log transformed mean fluorescence intensity for HEK 293 T cells treated as described above (n = 3). Cells were incubated for 72 h after transfection. Bars represent mean + SD where ****p < 0.0001

Polyplex solutions were mixed with CPC particulates and centrifuged before being used to transfect HEK 293 T cells. Cells treated with the supernatant resulting from the centrifugation of the mixture of polyplex solution and CPC particulates (CPC/Poly Supernatant) exhibited no detectable transfection, while cells treated with the resuspended pellet of CPC particulates resulting from the centrifugation of the mixture (CPC/Poly Mixture) exhibited minimal transfection efficiency (0.35%) (Fig. 4). Cells treated with either uncentrifuged polyplex solution (Polyplexes) or centrifuged polyplex solution (Polyplex Supernatant) exhibited equal levels of transfection (Fig. 4).

## Discussion

### Characterization of CPC prints

While CPC formulations typically use calcium phosphate particulates suspended in water, we opted for a newer formulation that uses an oily carrier fluid and thus does not begin hardening until it is exposed to water [54]. Prior work already established that this CPC formulation can be adequately hardened via either immersion in water (termed the “Water Alone”, or “WA” method in this article) or incubation in a highly humid environment (termed the “Vapor Alone”, or “VA”, method in this article), but it was noted that the WA method led to the formation of cracks in the strands that negatively impacted the prints’ applicability by reducing its mechanical strength [55]. Additionally, when we observed the surface of the implant hardened with the VA method with scanning electron microscopy, we found that the surface appeared to have less texture than the surface from the implant hardened with the WA method (Fig. 1B(iv)). This is important because research investigating bone cell differentiation on the surface of implants made of titanium [11, 12], polymers [56, 57], and calcium phosphate [58, 59] has shown that having more textured implant surfaces results in greater bone cell differentiation. As a result of this prior work, we aimed to combine the higher mechanical strength of the VA method with the greater surface texture of the WA method by taking prints initially hardened via the VA method and subjecting them to secondary incubations. For these additional incubations, the prints were immersed in either ultrapure water (termed the “vapor + water”, or “VW”, method in this article) or simulated body fluid (SBF) (termed the vapor + SBF”, or “VS”, method in this article) (Fig. 1A). When we observed the prints under high magnification, we found that a more textured surface was visible in the VW and VS prints when compared to the VA prints (Fig. 1B(iv-vi)), though the surfaces still appeared less textured than those of the WA method. This visual difference in surface texture was confirmed by SSA analysis of printed CPC rods that were subjected to differing hardening methods (Fig. 2A). Rods hardened with the VW and VS methods produced surfaces that had a higher SSA than those hardened with the VA method, confirming that they had a more textured surface, though the SSA values were lower than that of the WA method. By visual appearance alone it seems that the WA group should indeed have the highest SSA, though we believe that the SSA value for the WA group is artificially inflated because we cannot distinguish the nitrogen adsorbed to the surface exposed by the cracks from the nitrogen adsorbed to the exterior surface.

We assessed the compressive strengths of CPC lattices hardened via the WA, VA, VW, and VS methods to determine which hardening method produced lattices with the best mechanical properties. We found that lattices hardened with the VS method had higher compressive strength and Young’s modulus values than those hardened with the WA and VA methods (Fig. 2B and C). This increase in mechanical strength was likely induced by mineralization within the interior of the strands of the CPC lattices. Greater mechanical strength is important for bone regenerative implants because it makes it less likely for an implant to get damaged or deformed by forces exerted on the implant from the implantation procedure and/or from local tissue.

The CPC we used is known to transform into calcium deficient hydroxyapatite during hardening [55]. While this phase of calcium phosphate is similar to hydroxyapatite, the qualifier “calcium deficient” means that the material will absorb calcium from its surroundings until it becomes “stoichiometric” hydroxyapatite [60–63]. Specifically, when calcium deficient hydroxyapatite is immersed in a solution containing calcium (such as cell culture medium), calcium from the surroundings will be absorbed, phosphate will be released, and the medium will become more basic as a result of the released phosphate [60]. This phenomenon has been linked to a decline in the viability of cells incubated in static culture with calcium deficient hydroxyapatite materials [60–62]. Based on these findings, we decided to measure calcium content and pH of medium after 24 h of incubation with 1 mm by 7.5 mm discs to assess the ion flux caused by our scaffolds. We found that CPC scaffolds hardened via all hardening methods reduced the calcium content to less than 50% of the content of DMEM and increased medium pH relative to DMEM (Fig. 2D and E). The magnitude of calcium reduction and pH increase varied between the CPC hardening methods, with the VA scaffolds having both the greatest decline in calcium content and the greatest increase in medium pH. We believe this occurred because the VA scaffolds were not immersed in any liquid medium at any time during preparation, and thus the ions within the scaffold were unable to dissolve and reprecipitate into more thermodynamically stable calcium phosphate crystals [64]. However, the increase in pH observed after incubation of CPC materials should enhance bone formation, since it has been reported that slightly alkaline environments enhance bone cell activity while acidic conditions impair bone cell activity [65–67].

We assessed osteogenic differentiation of BMSCs seeded onto CPC discs by measuring the ALP activity. We found that the ALP activities from our different hardening methods followed the same pattern as the mechanical testing results: WA < VA < VW < VS. While the VW and VS groups were not significantly different from each other and both showed significant differences from the WA and VA groups, the VS group had a greater mean ALP activity than the VW group. From this we concluded that the differing hardening methods produce differences in differentiation, with the VS method seeming to produce the most differentiation. Interestingly, the WA group had the lowest ALP activity of all groups, despite it having the highest SSA. This may be due to two phenomena: the effect of medium pH on osteoblast differentiation/proliferation and the impact of surface texture morphology. Firstly, as noted above, slightly alkaline environments have been shown to increase their ALP activity [65–67]. Our data shows that CPC hardened with the VA, VW, and VS methods increase the pH of DMEM more than the WA method does, and thus the lesser increase in pH from the WA CPC may not have stimulated bone cell differentiation as much as the other CPCs hardened with the VA, VW, and VS methods. In addition, calcium phosphate surfaces with “needle-like” nanocrystals on the surface have been shown to outperform surfaces with “plate-like” nanocrystals on the surface in terms of BMSC osteogenic differentiation and bone regeneration in vivo [13, 58, 60]. The CPC hardened with the WA method clearly has “plate-like” nanocrystals on its surface while the VW and VS surfaces more closely resemble “needle-like” nanocrystals (Fig. 1B), and thus this difference in surface texture morphology may have also contributed to poor differentiation of BMSCs seeded onto CPC materials hardened with the WA method. However, in vitro conditions are quite different from the bone healing environment in vivo. As such, in future work CPC scaffolds hardened with our different hardening methods should be compared in an animal bone defect model to confirm whether the differences in osteogenic differentiation observed in vitro produce different bone healing outcomes in vivo.

### Characterization of gene expression from gene-activated CPC discs and lattices

For our preliminary CPC gene-activation experiments we printed discs made of CPC (7.5 mm in diameter, 1 mm thick) and gene-activated them using a lyophilized coating method drawn from earlier work by Laird et al. [53] and Malkawi et al. [68], wherein polyplexes were mixed with a concentrated lyoprotectant solution prior to being pipetted onto the surface and lyophilized. In the present study, sucralose (final concentration of 2% w/v) was used as the lyoprotectant for polyplexes and cells were seeded directly onto the CPC discs. When we compared the transfection efficiencies resulting from different hardening methods, we found that the VW and VS hardening methods produced the highest mean transfection efficiencies (13.42% and 12.97%, respectively), though they had very high variability (Fig. 3A and B). These results resemble prior findings from Choi et al. [69] involving a lipofectamine-based gene-delivery system loaded onto mineral-coated polymer materials. Choi et al. found that, by modulating the ion content of the mineral coating solution they used to coat polymer surfaces, they could influence the coating’s morphology, calcium dissolution, and transfection after being loaded with lipofectamine-pDNA complexes. They attributed the difference in transfection to the difference in release of calcium from their coatings, since greater soluble calcium concentration has been shown to enhance non-viral transfection [70–72]. Since our prints absorb calcium from cell culture medium, the transfection of cells by our polyplexes may have been increasingly impaired as calcium was removed from the cell culture medium during incubation.

For our follow-up CPC gene-activation experiments, we printed CPC lattices rather than discs so as to assess the gene expression from scaffolds that more closely resemble the types of scaffolds likely to be implanted into a bone defect (i.e. a scaffold with an internal pore network) [13, 24, 55]. As a result of the higher mean transfection, superior mechanical strength, reduced calcium absorption, and superior BMSC differentiation, we chose to use the VS hardening method for our characterization of gene expression from gene-activated CPC lattices. We then assessed both transfection efficiency and the gene expression profile in a model cell line (HEK 293 T cells) and an osteoblast precursor cell type (MC3T3 cells). In contrast to the moderate transfection efficiencies from gene-activated discs (~ 13% for VW and VS methods), the transfection efficiencies from gene-activated lattices were very low for both HEK 293 T cells (1.137%) and MC3T3 cells (1.273%) (Fig. 3C and F). When we assessed the gene expression profile from cells seeded onto gene-activated CPC lattices over the course of 8 days, we found that the gene-activated lattices were able to induce some gene expression beginning at day 2 and ending at day 8, though the expression level was low in both HEK 293 T cells and MC3T3 cells (Fig. 3E and H). In particular, the HEK 293 T positive control group had vastly higher expression of luciferase despite being treated with an equal dose of polyplexes, reaching a peak luciferase expression that was 10 times higher than the peak for the gene-activated CPC lattices. These results show that while the gene-activation process does produce measurable gene expression, the level of gene expression from gene-activated CPC lattices should be improved to ensure that they can induce gene expression of osteogenic proteins at levels that will improve bone healing.

### Determination of source for low expression

Since there was a large difference in the transfection efficiency between gene-activated CPC discs and gene-activated CPC lattices, we hypothesized that this might be due to polyplex adsorption to the ionic surface of the CPC. The lattices have a greater surface area for polyplex adsorption relative to the discs (225.97 mm^2^ for lattices vs. 111.92 mm^2^ for discs), and so the decline in transfection efficiency could be due to a greater number of polyplexes adsorbing to the CPC lattice surface, preventing them from diffusing through the cell culture medium and transfecting cells. To test this hypothesis we pulverized CPC prints to create fine particulates with a large total surface area for polyplexes to adsorb to. When we exposed polyplexes to the particulates then centrifuged the particulates out of solution, we found that the polyplexes seem to have been removed from the solution entirely, since the resulting supernatant was incapable of transfecting cells (Fig. 4). However, there was minimal, but detectable, transfection in cells treated with the particulates themselves, demonstrating that functional polyplexes remained adsorbed to the CPC particulates. In contrast, polyplex solutions that were centrifuged but not exposed to CPC particulates were able to transfect cells at an equal transfection efficiency to polyplex solutions that were not centrifuged at all. From these results we concluded that the increase in surface area that polyplex solutions were exposed to when loaded onto CPC lattices explains the decline in transfection seen when we transitioned from CPC discs to CPC lattices.

## Conclusion

Herein we have demonstrated that we can produce a 3D printed, surface modified, gene-activated CPC scaffold that can successfully transfect multiple cell types seeded onto its surface. However, despite successful transfection in seeded cells, the level of gene expression from 3D printed lattices is very low and thus inducing expression of BMP-2 with this current system is unlikely to lead to enhanced bone formation in an in vivo bone regeneration model. Based on the data presented here, it seems that an increase in the total surface area of the CPC material exposed to polyplex solutions produces a reduction in transfection via adsorption of polyplexes to the CPC surface. Gene-activated scaffolds represent a promising alternative to current bone regeneration methods involving BMP-2 since they may avoid the issues of high costs and ectopic bone formation associated with current use of BMP-2 protein in the clinic. Future work should explore methods to increase the transfection efficiency from 3D printed CPC lattices, such as modifying the surface properties of prints to reduce adsorption or by using a different gene-delivery system less prone to surface adsorption to charged surfaces. By coating CPC prints in modified SBF solutions that are aimed at producing surfaces that enhance transfection from cationic gene-delivery systems, a CPC scaffold may be able to produce BMP-2 at a concentration that may enhance bone healing. Alternatively, switching from cationic gene-delivery systems (like the polyplexes described here) to neutrally charged lipid nanoparticles may circumvent the issue of adsorption to CPC surfaces.

## Data Availability

The corresponding author will provide the datasets created and/or analyzed during the current study upon reasonable request.

## Abbreviations

BMP-2: Bone morphogenetic protein 2
CPC: Calcium phosphate cement
PEI: Polyethylenimine
pDNA: Plasmid DNA
SBF: Simulated body fluid
BMSC: Bone marrow-derived mesenchymal stem cells
ALP: Alkaline phosphatase
